# Assessing urinary flow rate, creatinine, osmolality and other hydration adjustment methods for urinary biomonitoring using NHANES arsenic, iodine, lead and cadmium data

**DOI:** 10.1186/s12940-016-0152-x

**Published:** 2016-06-10

**Authors:** Daniel R. S. Middleton, Michael J. Watts, R. Murray Lark, Chris J. Milne, David A. Polya

**Affiliations:** School of Earth, Atmospheric and Environmental Sciences & Williamson Research Centre for Molecular Environmental Science, University of Manchester, Oxford Rd, Manchester, M13 9PL UK; Inorganic Geochemistry, Centre for Environmental Geochemistry, British Geological Survey, Keyworth, Nottinghamshire, NG12 5GG UK

**Keywords:** Biomonitoring, Hydration adjustment, Creatinine, Osmolality, Urinary flow rate, NHANES

## Abstract

**Background:**

There are numerous methods for adjusting measured concentrations of urinary biomarkers for hydration variation. Few studies use objective criteria to quantify the relative performance of these methods. Our aim was to compare the performance of existing methods for adjusting urinary biomarkers for hydration variation.

**Methods:**

Creatinine, osmolality, excretion rate (ER), bodyweight adjusted ER (ERBW) and empirical analyte-specific urinary flow rate (UFR) adjustment methods on spot urinary concentrations of lead (Pb), cadmium (Cd), non-arsenobetaine arsenic (As^IMM^) and iodine (I) from the US National Health and Nutrition Examination Survey (NHANES) (2009–2010 and 2011–2012) were evaluated. The data were divided into a training dataset (*n* = 1,723) from which empirical adjustment coefficients were derived and a testing dataset (*n* = 428) on which quantification of the performance of the adjustment methods was done by calculating, primarily, the correlation of the adjusted parameter with UFR, with lower correlations indicating better performance and, secondarily, the correlation of the adjusted parameters with blood analyte concentrations (Pb and Cd), with higher correlations indicating better performance.

**Results:**

Overall performance across analytes was better for Osmolality and UFR based methods. Excretion rate and ERBW consistently performed worse, often no better than unadjusted concentrations.

**Conclusions:**

Osmolality adjustment of urinary biomonitoring data provides for more robust adjustment than either creatinine based or ER or ERBW methods, the latter two of which tend to overcompensate for UFR. Modified UFR methods perform significantly better than all but osmolality in removing hydration variation, but depend on the accuracy of UFR calculations. Hydration adjustment performance is analyte-specific and further research is needed to establish a robust and consistent framework.

**Electronic supplementary material:**

The online version of this article (doi:10.1186/s12940-016-0152-x) contains supplementary material, which is available to authorized users.

## Background

Urinary biomonitoring is the preferred method of exposure and nutritional assessment for many chemical elements and metabolites given its non-invasiveness, logistical appeal and ease of measurement with modern analytical techniques [1]. This is true for potentially harmful elements like arsenic (As) [2], essential nutrients like iodine (I) [3] and drug and organic compounds [4, 5], making the meaningful interpretation of urinary data a requirement with implications for public health, occupational health and forensic applications. The US National Health and Nutrition Examination Survey (NHANES) has proved an invaluable, growing resource of chemical biomonitoring data [6], but the value of such data depend on their correct interpretation [7], challenges with which are currently limiting the full potential of urinary chemical biomarkers [6].

Urinary analyte concentrations are susceptible to variation from factors extending beyond exposure and are categorised [1] as follows: (i) time of sampling relative to exposure; (ii) inter-individual toxico-kinetic factors and (iii) physiological characteristics of the biomonitoring matrix. While the first two factors should not be ignored, the third, specifically the variation in dilution among spot urine samples, is addressed here.

While collection of 24 hr urine samples is preferred, it is not feasible for large biomonitoring studies due to resource limitations, cumbersome sample nature and volunteer compliance issues [8]. First morning void (FMV) or spot collections are common substitutes, but are limited in that they reflect the hydration status of the individual at the time of collection thus may differ markedly in dilution as a result of differences in urinary flow rate (UFR). Spot/FMV samples are nevertheless widely deemed acceptable provided that the effect of sample dilution is quantified and appropriately adjusted [9]. Several methods for adjusting spot/FMV data are currently employed but there is no consensus on which is the most appropriate.

The most common technique employed is creatinine adjustment, whereby urinary analyte concentrations are ratioed to creatinine concentrations. This method implicitly assumes that urinary creatinine is excreted at a constant rate and varies only as a function of UFR. However, these assumptions are of questionable validity, since creatinine concentrations have been shown to depend upon all of demographic group [10], protein intake [11], muscle mass [12] and malnutrition [13].

Alternative methods such as specific gravity (SG) or osmolality adjustment are commonly reported. Close agreement has been demonstrated between these methods [14] but osmolality, as measured by osmometry, has been described as the definitive measure of urinary concentration [15] despite being previously considered prohibitively expensive [16]. Osmometry is not susceptible to the same interferences as SG, conventionally measured by refractometry, which may be confounded in subjects with, for example, proteinuria and glucosuria. Urinary osmolality was a post-2008 inclusion in NHANES and, while similar factors affecting creatinine excretion were found to be responsible for variation in urinary osmolality, less influence was observed on osmolality than on creatinine in the US population [8].

Creatinine, SG and osmolality are all surrogate estimators of UFR with various degrees of effectiveness [17, 18]. Direct UFR measurements are now included in post-2008 NHANES cycles, with UFR determined as follows:

1 $$ UFR = V/t, $$

where *t* is the time elapsed between two urine voids and *V* is the volume of the second void. Excretion rates (ER), typically expressed in ng/hr, of the analyte can be calculated:

2 $$ ER = \left({C}_{vol}\times V\right)/t, $$

where *C*_*vol*_ is the measured, volume-based urinary analyte concentration, typically in nanograms per millilitre, *V* is volume, typically in millilitres and *t* is time, typically in hours. Additionally, bodyweight (BW) adjusted ER (ERBW), typically expressed in ng/kg-hr, can be calculated as follows:

3 $$ ERBW = \left({C}_{vol} \times V\right)/\left(t \times BW\right). $$

Adjusting urinary biomonitoring results according to Eqs. 2 and 3 was recently proposed to directly account for hydration status and address demographic variations in UFR more effectively than creatinine and osmolality adjustments [19]. While UFR, providing accurate measurement of time and volume, is more reflective of hydration status than surrogate measures, such as creatinine and osmolality, its application in Eqs. 2 and 3 directly incorporates hydration bias into results. Given that ERs can still apply to restricted time periods, they are dependent on UFR, i.e. hydration, at that time. Strong positive Spearman’s correlation coefficients (r_s_) have been reported between ERs of urinary analytes and UFR [20, 21], indicating that the adjustment in Eq. 2 is not theoretically robust.

Specifically, application of Eq. 2 implicitly assumes that analyte concentrations vary inversely proportionally with UFR. However, this was disputed by Araki et al. (1990), who observed analyte-specific, log-linear relationships between analyte concentrations and UFR of the form:

4 $$ log\ {C}_{vol} = a-b\  log\ UFR, $$

where *a* and *b* (referred to here as Araki’s *b* value) are analyte-dependent, empirically determined regression coefficients.

Araki et al. (1990) therefore proposed a modified UFR adjustment whereby analyte concentrations were adjusted to a standard UFR of 1 mL/min:

5 $$ {C}_{UFR}{\hbox{-}}_{adj} = {C}_{vol} \times UF{R}^b, $$

where Araki’s *b* values were derived for a number of analytes using multiple voids from single individuals subjected to water loading and water restrictive conditions. This UFR adjustment was found to be more effective than ER, creatinine and SG adjustment in removing UFR-dependent variation from adjusted urinary analyte concentrations [20, 21]. For datasets, such as NHANES, that do not contain extensive analyte concentration data for multiple voids from single individuals, a previously reported iterative method [22] may be used to calculate population-level Araki’s *b* values by optimising appropriate performance criteria.

Comparing the performance of urinary hydration adjustment methods requires assessment criteria appropriate to the needs of a given study. Suggested criteria are summarised in Table 1. For this work, the primary assessment criterion was the extent of removal of systematic dependence on UFR of adjusted urinary analyte concentrations (Criterion A, Table 1) as has been used previously [20, 22, 23]. Additionally, since blood biomonitoring is the preferred measure of exposure for several chemicals, notably lead (Pb), and is not as susceptible to the same level of variation as urinary concentrations [24], the correlation between adjusted urinary concentrations and blood concentrations was also used as a secondary assessment criterion (Criterion B, Table 1) and has been previously explored [25] for Pb. Similarly, in the case of cadmium (Cd), blood is a biomarker of both recent and cumulative Cd exposure, and urinary concentrations reflect cumulative exposures and Cd levels in the kidney [26]. Agreement between urinary and blood Cd concentrations have been reported [27], making it reasonable to hypothesize that the effective removal of hydration variation from spot urine samples may strengthen this relationship. Other possible assessment criteria include agreement with 24 hr excretion rates or composite concentrations, used previously [14], but the lack of 24 hr data in the NHANES survey does not permit this. Lastly, an independent external measure of exposure, e.g. drinking water analyte concentrations [28] could be used as an assessment criterion, but was not used in this study because of a lack of appropriate environmental data in NHANES.

**Table 1:** Suggested criteria for assessing performance of urinary biomonitoring adjustment methods

Criterion	Description	Performance metric
A	Correlation between adjusted spot analyte concentrations and UFR.	Weaker correlations indicating good performance.
B	Correlation between adjusted spot analyte concentration and an independent measure of internal dose, e.g. analyte concentration in blood.	Stronger correlations indicating good performance*.*
C	Correlation of spot analyte concentrations with analyte excretion over 24 hr or composite 24 hr concentrations.	Closer agreement/lower variation in spot samples indicating good performance*.*
D	Correlation of spot analyte concentration with an independent measure of/proxy for external exposure e.g. drinking water analyte concentration.	Stronger correlations indicating good performance*.*

Criteria A and B are used in this study

## Methods

### Aims

This paper aims to compare the performance of urinary biomonitoring hydration adjustment techniques using NHANES (2009–2010, 2011–2012) spot urinary concentrations of selected chemical analytes and, in particular, test whether or not analyte-specific UFR adjusted concentrations provide a more robust adjustment than creatinine, osmolality, ER or ERBW adjustments. Arsenic and iodine were selected as chemicals on which to make these tests as respectively toxic and essential elements for which urinary biomonitoring is widely used. Additionally, Pb and Cd were selected for study, due to the availability of paired urine-blood samples in the NHANES database and the applicability of criterion B to these elements. This provides the opportunity to compare the adjustment performance characteristics of two independent assessment criteria.

### Data acquisition

Data from the NHANES 2009–2010 and 2011–2012 surveys were acquired from the NHANES website [29]. Volunteer consent information and dataset access can be found online: http://www.cdc.gov/nchs/nhanes.htm. Data on demographic variables; body measurements; standard biochemistry profile; diabetes; kidney conditions; plasma fasting glucose; urinary flow rates; urinary creatinine; urinary osmolality; urinary metals; total and speciation urinary As; urinary I and blood metals were downloaded in SAS (.xpt) format. Data were converted to MS Excel (.xlsx) format using the R programming environment SASxport and xlsx packages [30, 31], before being matched by sequence number (SQN) in MS Access. Volunteers with data present on gender, age, bodyweight, urinary creatinine, urinary osmolality, UFR, As speciation and blood metals, of either non-Hispanic white, non-Hispanic black or Mexican American ethnicity were initially included.

Volunteers with evidence of health conditions that could affect the performance of urinary adjustment calculations were excluded using previously published criteria [10, 19]: urinary albumin-creatinine ratio >30 mg/g creatinine was treated as albuminuria and diabetics were identified by self-reported physician diagnosis or plasma glucose ≥126 mg/dL (≥8 h fasting) or ≥200 mg/dL (<8 h fasting). Chronic kidney disease (CKD) was identified by self-reported physician diagnosis or an estimated glomerular filtration rate (eGFR) <60 mL/min/1.73 m^2^ using the Chronic Kidney Disease Epidemiology Collaboration (CKD-EPI) equation [32]. For volunteers aged <18, eGFR was estimated using the Bedside Schwartz equation [33]. Finally, volunteers without detectable concentrations of urinary Pb, Cd, Total As and I were excluded to limit the effects of censoring on analyses.

Additional data from a single volunteer consisting of multiple spot Cd concentrations and UFR measurements were reproduced [34] for observational purposes only.

### Analytical measurements

Detailed analytical methodologies for the analytes investigated in this paper, plus other analytical components of NHANES, are reported online: http://www.cdc.gov/nchs/nhanes/nhanes2011-2012/lab_methods_11_12.htm. Urine samples were provided by volunteers at the NHANES mobile examination center (MEC). Volunteers were asked to fully void the bladder and report the last time of previously doing so. Volumes of the samples provided at the MEC were measured and used, with the previous void times reported by volunteers, to calculate UFR as per Eq. 1. For volunteers with initial urinary volumes below requirement, subsequent voids were collected and composite UFRs were calculated using the total volumes and times covered by all voids. This ensured that laboratory measurements made on pooled samples consisting of multiple voids corresponded to the correct UFRs. Urinary osmolality was measured using freezing-point depression (cryoscopic) osmometry performed with an Osmette II, Model 5005 Automatic Osmometer (Precision Systems Inc.). Urinary creatinine was determined using an enzymatic (creatininase) reaction and a Roche/Hitachi Modular P Chemistry Analyzer. Urinary total I, Pb and Cd and whole blood Pb and Cd were determined using inductively coupled plasma dynamic reaction cell mass spectrometry (ICP-DRC-MS) (PerkinElmer ELAN® 6100 DRCPlus or ELAN® DRC II). Urinary As speciation was performed using high performance liquid chromatography (HPLC) coupled to ICP-DRC-MS. Combined urinary inorganic As and methylated metabolites (As^IMM^) was calculated as the sum of arsenous acid (As^III^), arsenic acid (As^V^), monomethylarsonic acid (MMA) and dimethylarsonic acid (DMA) species, as this is the routine biomarker of As exposure and does not incorporate non-toxic arsenobetaine. Osmolality and UFR were both determined at the MEC shortly after urine collection. The remaining measurements were made after urine and blood samples had been frozen at -20 °C and shipped to relevant laboratories where they remained frozen until analysis to prevent evaporation and the inter-conversion of As species.

### Urinary analyte adjustment calculations

Data were read into the R programming environment [35] and partitioned into two subsets by simple random sampling using the caret package [36]:

1. **Training dataset**: 80 %, reserved for Araki’s *b* value derivation.
2. **Testing dataset**: 20 %, for applying and assessing the performance of adjustment calculations.

The partition of 80:20 % was deemed suitable [37] and was selected to (i) ensure sufficient training data were available; (ii) retain a testing dataset of a size comparable to that of a biomonitoring study in which these kinds of adjustments may be employed and (iii) to preserve the distribution of demographic and analytical variables between both datasets.

Urinary analyte excretion rates (ER, ng/hr) and bodyweight adjusted excretion rates (ERBW, ng/kg-hr) were calculated using Eqs. 2 and 3, respectively [19]. Conventional creatinine-adjusted analyte concentrations were expressed in μg/g creatinine as follows:

6 $$ {\mathrm{C}}_{\mathrm{cr}\hbox{-} \mathrm{a}\mathrm{d}\mathrm{j}} = {\mathrm{C}}_{\mathrm{vol}}/{\mathrm{C}}_{\mathrm{cr}}, $$

where C_cr_ is the specimen creatinine concentration in grams per litre. Osmolality adjustment was performed using an equation based on the Levine-Fahy specific gravity adjustment [38] as follows:

7 $$ {\mathrm{C}}_{\mathrm{osm}\hbox{-} \mathrm{a}\mathrm{d}\mathrm{j}} = {\mathrm{C}}_{\mathrm{vol}} \times \left({\mathrm{Osm}}_{\mathrm{ref}}/{\mathrm{Osm}}_{\mathrm{meas}}\right), $$

where, for consistency with recent publications [19], Osm_ref_ is the median osmolality (mOsm/kg) of training data volunteers (734 mOsm/kg) and Osm_meas_ is that measured in the individual specimen. Araki’s *b* values were extracted using an adaption of a previously published approach [22] which involved a simple numeric method. Pearson correlation coefficients (r_p_) for criterion A and criterion B were calculated from the training data for values of Araki’s *b* from 0 to 1.5 at intervals of 0.01. Araki’s *b* values that yielded optimum correlations for criterion A (minimizing absolute value of r_p_) and separately for criterion B (maximizing r_p_) were determined. Araki’s *b* values were also derived on demographic subsets of specific age groups and specific genders/ethnicities to identify patterns between groups. Optimum Araki’s *b* values were used to perform Araki’s modified UFR adjustment (Eq. 5), henceforth referred to as UFRA and UFRB when adjusted using optimum Araki’s *b* values for criteria A and B, respectively. An R script has been provided in Additional file 1 to allow other groups to derive Araki’s *b* values and perform hydration adjustments.

### Statistical analyses

Due to the specific application of NHANES data in assessing adjustment methods rather than making inferences of biomonitoring measurements in the US population, sample weights were not incorporated into analyses. Statistical tests (and graphical presentations) were performed using R version 3.0.0 (base package) [35]. Urinary and blood analyte data were positively skewed and, therefore, geometric means (GM) were calculated as opposed to arithmetic means. For the same reason, Pearson correlations of urinary analyte concentrations against UFR and blood analyte concentrations were calculated on natural log (ln) transformed data with significance tests (*p*-values) and 95 % confidence intervals (CI) using the ‘cor.test’ function. Pearson’s, as opposed to Spearman’s, correlation was selected to prevent the loss of information that occurs when data are reduced to ranks in the process of calculating Spearman’s correlation. It was necessary to test the significance of the difference between correlations of, for example, urinary Pb with blood Pb adjusted by different methods. These correlations are not independent (because of the common variable, blood Pb), so the Williams’s test [39] was performed using the r.test function in the psych package [40]. Point density contour lines were added to plots using two-dimensional kernel density estimation in the MASS package [41].

## Results

### Exploratory analyses – training data

Inclusion of data for volunteers with the appropriate demographic, examination and laboratory variables yielded records for 3539 individuals. This was reduced to 2668 following the exclusion of volunteers with evidence of albuminuria, diabetes or CKD. A reduction to 2151 records was made after excluding those with urinary analyte concentrations below analytical detection limits. These 2151 records were partitioned independently and at random into a training dataset of 1723 records and a testing dataset of 428 records reserved for independent adjustment comparisons. Study group characteristics, GMs and ranges of creatinine osmolality, UFR, unadjusted urinary As^IMM^, I, Pb Cd and blood Pb and Cd of training and testing datasets are shown in Table 2, demonstrating the preservation of characteristic and analyte distributions following the partitioning of the data.

**Table 2:** Demographic characteristics and unadjusted analyte geometric means (GM) and ranges for training and testing datasets

	Training data	Testing data
Demographic group, *n* (%)		
All	1,723	428
Male	887 (51)	243 (57)
Female	836 (49)	185 (43)
Non-Hispanic white	841 (49)	216 (51)
Non-Hispanic black	489 (28)	116 (27)
Mexican American	393 (23)	96 (22)
6–11 years old	148 (8)	39 (9)
12–19 years old	268 (16)	63 (15)
20–39 years old	544 (32)	125 (29)
40–59 years old	461 (27)	120 (28)
>60 years old	302 (17)	81 (19)
Analytical measurement, GM (range)		
Creatinine, g/L	1.1 (0.1–8)	1.1 (0.09–5.6)
UFR, mL/min	0.7 (0.03–34.5)	0.7 (0.06–5.5)
Osmolality, mOsm/kg	635 (91–1,394)	630 (84–1,350)
Urinary As^IMM^, μg/L	6.3 (2.9–386)	6.4 (2.9–105)
Urinary I, μg/L	149 (8–15,651)	161 (16.9–9,322)
Urinary Pb, μg/L	0.5 (0.08–49.6)	0.5 (0.08–14.3)
Urinary Cd, μg/L	0.2 (0.04–6.2)	0.2 (0.04–4.8)
Blood Pb, μg/dL	1.1 (0.2–33.7)	1.1 (0.2–22)
Blood Cd, μg/L	0.3 (0.1–8.7)	0.3 (0.1–4)

**Fig. 1:**
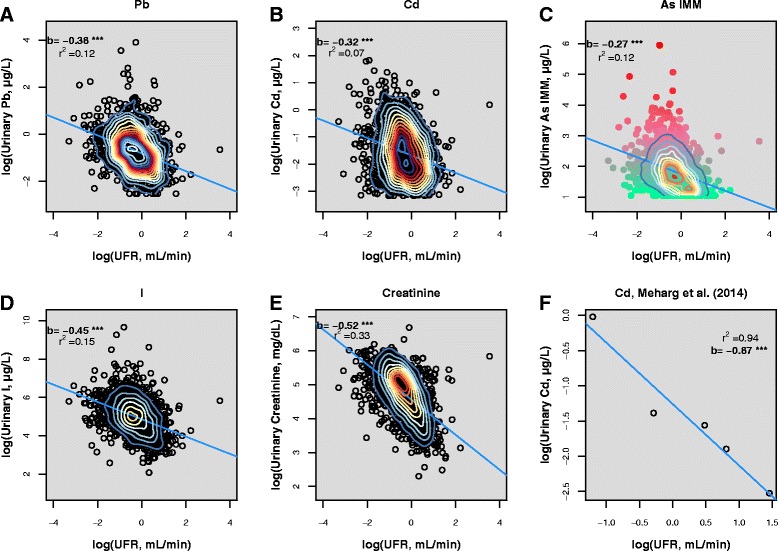
Unadjusted urinary Pb (**a**), Cd (**b**), As^IMM^ (**c**), I (**d**) and creatinine (**e**) plotted against UFR (NHANES 2009–2012 (CDC, 2015) training data). Multiple spot Cd measurements (Meharg et al., 2014) from a single volunteer are shown for comparison (**f**). Linear regression lines (*blue*) are displayed with regression slopes and r^2^ values. *** denotes significance to p < 0.001. Point density contours were plotted using two-dimensional kernel density estimation. In (**c**), the transition from *green* to *red* depicts increasing concentration of urinary dimethylarsonic acid (DMA)

Training dataset log transformed urinary analytes, including creatinine, showed (Fig. 1) significant (*p* < 0.001) negative, log-linear relationships with UFR. This confirmed previous findings [20]. The weak (Pb, Cd, As^IMM^ and I in Fig. 1a-d) to moderate (creatinine, Fig. 1e) r^2^ values indicated that the majority of variation in analyte concentrations were not explained solely by UFR – this was most pronounced for Cd and least pronounced for creatinine. Large variations in urinary analyte concentration relative to variations in UFR would be expected to result in criterion A Araki’s *b* values >1, however the calculated Araki’s *b* values for all urinary analytes were substantially <1, indicating other controls on urinary analyte concentrations. Notably, for As, the relationship between urinary As^IMM^ and UFR was particularly impacted by the range of concentrations of DMA (Fig. 1c). It is noteworthy that the Araki’s *b* value calculated from data for a single individual [34] for Cd (Fig. 1f) (0.87) is substantially different from that calculated data from multiple individuals (0.32).

### Derivation of Araki’s b values – training data

Araki’s *b* values derived in the present study from pooled NHANES data (single voids from multiple individuals) by optimising criterion A or criterion B are presented in Table 3 along with published values (where available) from mean data derived previously [20, 21] (multiple voids from single individuals). The criterion A optimised Araki’s *b* values for Pb (0.38), Cd (0.32) and creatinine (0.52) were all somewhat lower than values reported previously [20, 21]. The criterion B optimised values for Pb (0.56) and Cd (0.62) were closer to those published previously [20, 21]. We note that the Araki’s *b* values derived to optimise Criterion A agreed with the *b* values that describe the slopes of the linear relationships shown in Fig. 1.

**Table 3:** Araki’s *b* values derived for Pb, Cd, As^IMM^ and I in the present study (NHANES 2009–2012 (CDC, 2015) training dataset) compared with previously reported literature values

Analyte	Araki’s *b* value			
	Criterion A (UFR) optimised (present study)	Criterion B (Blood) optimised (present study)	Araki et al. (1986)^a,c^	Araki et al. (1990)^b,c^
Pb	0.38	0.56	0.50 (0.45–0.91)	0.45 (0.39–0.54)
Cd	0.32	0.62	-	0.58 (0.42–0.66)
As^IMM^	0.27	-	-	-
I	0.45	-	-	-
Creatinine	0.52	-	0.87 (0.67–1.01)	0.68 (0.58–0.75)

^a^ Mean value of 10 subjects derived by linear regression

^b^ Mean value of 4 time quadrants derived by linear regression

^c^ Range of significant values in parentheses

**Fig. 2:**
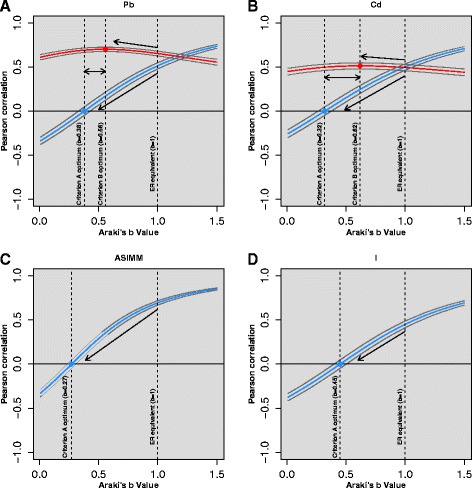
Sensitivity of Pearson correlations to Araki’s *b* value for NHANES 2009–2012 (CDC, 2015) training data for Pb (**a**), Cd (**b**), As^IMM^ (**c**) and I (**d**) for criterion A (urinary analyte versus UFR, *blue lines*) and criterion B (urinary analyte versus blood analyte, *red lines*) with 95 % confidence intervals (*grey lines*). Optimum criterion A (*blue diamonds*) and criterion B (*red diamonds*) Araki’s *b* values are displayed and, in the case of Pb and Cd, the difference between these values is highlighted by *double-headed arrows*. *Single-headed arrows* illustrate the improvement in criterion A (decreasing correlation) and criterion B (increasing correlation) correlations relative to the equivalent Araki’s *b* value implicit of ER

The sensitivity of Pearson correlations to model Araki’s *b* values for criteria A (Pb, Cd, As^IMM^, I) and B (Pb, Cd) are illustrated in Fig. 2. In all cases, the criterion A and criterion B optimised *b* values were all lower than the *b* value (*b* = 1) implicit in conventional ER approaches and were all better adjustments based on using Pearson correlation as the metric.

### Comparison of adjustment methods – testing data

**Fig. 3:**
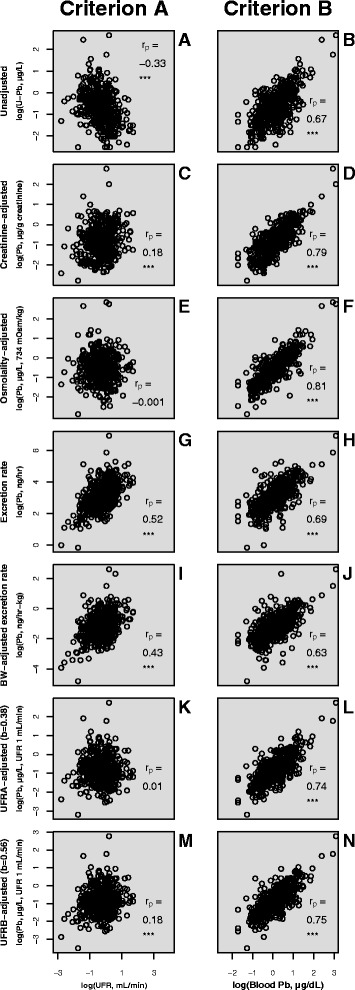
Scatterplots of unadjusted (**a**, **b**); creatinine adjusted (**c**, **d**); osmolality adjusted (**e**, **f**); ER adjusted (**g**, **h**); ERBW adjusted (**i**, **j**); UFR adjusted with Araki’s *b* optimised to criterion A (**k**, **l**) and criterion B (**m**, **n**) urinary Pb concentrations vs UFR (criterion A) (**a**, **c**, **e**, **g**, **i**, **k**, **m**) or blood Pb concentrations (criterion B) (**b**, **d**, **f**, **h**, **j**, **l**, **n**). Data: NHANES 2009–2012 (CDC, 2015) testing dataset. *** denotes significance to *p* < 0.001

The different adjustment methods were performed on analyte concentrations and the resulting GM concentrations and ranges are presented in Table 4. The performance of adjustment methods for urinary analyte concentrations from the testing dataset were assessed against criterion A (Pb, Cd, As^IMM^ and I) (Table 5) and criterion B (Pb and Cd) (Table 6). The relative performance of these methods, using Pb as an example, is also illustrated in Fig. 3 and summarized below:

**Table 4:** Geometric means (GM) and ranges of urinary analytes following adjustment by various methods (testing dataset)

Urinary analyte, GM (range)	Unadjusted, μg/L	Creatinine-adjusted, μg/g creatinine	Osmolality-adjusted, μg/L, 734 mOsm/kg	ER, ng/hr	ERBW, ng/hr-kg	UFRA, μg/L, UFR 1 mL/min	UFRB, μg/L, UFR 1 mL/min
As^IMM^	6.4 (2.9–105)	5.7 (1.3–55.6)	7.5 (2–82.4)	279 (18.5–3,921)	3.9 (0.2–64.8)	5.9 (2.2–92.4)	-
I	161 (16.9–9,322)	143 (23.3–4,615)	188 (28.6–8,661)	7,024 (558–352,368)	97.5 (9.6–4,199)	140 (24.2–7,572)	-
Pb	0.5 (0.08–14.3)	0.5 (0.06–16.1)	0.6 (0.06–17.5)	22.4 (0.8–1,042)	0.3 (0.008–13.9)	0.5 (0.04–15.4)	0.4 (0.03–15.9)
Cd	0.2 (0.04–4.8)	0.2 (0.03–2.6)	0.25 (0.03–4.9)	9.2 (0.5–97.5)	0.1 (0.006–1.5)	0.2 (0.02–3)	0.2 (0.02–2.2)

**Table 5:** Pearson correlations for performance Criterion A across the range of adjustment methods investigated for NHANES 2009–2012 (CDC, 2015) (testing dataset)

Adjustment method	r_p_ (95 % CI)					
	Pb		Cd		As^IMM^	I
Unadjusted	-0.33*** (-0.41, -0.24)		-0.25*** (-0.34, -0.26)		-0.37*** (-0.45, -0.28)	-0.39*** (-0.47, -0.31)
Creatinine	0.18*** (0.09, 0.27)	b	0.18*** (0.09, 0.27)	b	0.32*** (0.24-0.41)	0.09 (-0.001, 0.19)
Osmolality	**-0.001 (-0.10, 0.09)**	a	**0.02 (-0.07, 0.12)**	a	0.10* (0.003-0.19)	-0.09 (-0.18, 0.004)
ER	0.52*** (0.45, 0.59)		0.47*** (0.39, 0.54)		0.70*** (0.65, 0.74)	0.45*** (0.37, 052)
ERBW	0.43*** (0.35, 0.50)		0.42*** (0.34, 0.50)		0.60*** (0.53, 0.66)	0.35*** (0.26, 0.43)
UFRA	**0.01 (-0.08, 0.11)**	a	**-0.01 (-0.10, 0.09)**	a	**-0.03 (-0.12, 0.07)**	**-0.01 (-0.10, 0.09)**
UFRB	0.18*** (0.09, 0.27)	b	0.22*** (0.13, 0.31)	b	-	-

Correlations were calculated on natural log transformed data. *** and * denote significance to *p* < 0.001 and <0.05, respectively. Bold font denotes the best performing adjustment method for each analyte. Correlations share a letter when not significantly different from one another

**Table 6:** Pearson correlations for performance Criterion B across the range of adjustment methods investigated for NHANES 2009–2012 (CDC, 2015) (testing dataset)

Adjustment method	r_p_ (95 % CI)			
	Pb		Cd	
Unadjusted	0.67 (0.61, 0.72)	de	0.58 (0.51, 0.64)	d
Creatinine	**0.79 (0.75, 0.82)**	ab	**0.65 (0.59, 0.70)**	ab
Osmolality	**0.81 (0.78, 0.84)**	a	**0.66 (0.60, 0.71)**	a
ER	0.69 (0.63,0.73)	d	0.59 (0.52, 0.64)	dc
ERBW	0.63 (0.57, 0.68)	e	0.57 (0.50, 0.63)	d
UFRA	0.74 (0.70, 0.78)	c	0.62 (0.56, 0.67)	bc
UFRB	0.75 (0.70, 0.79)	bc	0.62 (0.56, 0.68)	b

Correlations were calculated on natural log transformed data. All correlations are significance to *p* < 0.001. Bold font denotes the best performing adjustment method for each analyte. Correlations share a letter when not significantly different from one another

*Criterion A*

- Pb: UFRA, Osmolality > Creatinine, UFRB > **Unadjusted** > ERBW > ER
- Cd: UFRA, Osmolality > Creatinine, UFRB > **Unadjusted** > ERBW > ER
- As^IMM^: UFRA > Osmolality > Creatinine > **Unadjusted** > ERBW > ER
- I: UFRA > Osmolality > Creatinine > ERBW > **Unadjusted** > ER

*Criterion B*

- Pb: Osmolality ≥ Creatinine ≥ UFRB ≥ UFRA > ER ≥ **Unadjusted** ≥ ERBW
- Cd: Osmolality ≥ Creatinine ≥ UFRB ≥ UFRA ≥ ER ≥ **Unadjusted** ≥ ERBW

Irrespective of whether criterion A or criterion B was used to assess adjustment method performance, it was evident that UFRA, UFRB, creatinine and osmolality adjustment methods all provided for a statistically significant improvement relative to unadjusted analyte concentrations. Of these, osmolality adjustment was determined to be the optimal adjustment method except for As^IMM^ and I for which UFRA showed a marginally better performance. The criterion A-based performance of osmolality and UFRA adjustments were equally good for Pb and Cd. Indeed, osmolality adjustment resulted in a weak (*r*_p_ = 0.10) significant (*p* < 0.05) correlation only in the case of osmolality adjusted As^IMM^ versus UFR. Creatinine adjustment, in contrast, yielded significant positive correlations with UFR for Pb (*r*_p_ = 0.18), Cd (*r*_p_ = 0.18) and As^IMM^ (*r*_p_ = 0.32). Iodine was an exception, with no significant correlation of creatinine adjusted I concentrations against UFR.

Excretion rate adjustment methods (ER, ERBW) performed worse than any of UFRA, UFRB, creatinine or osmolality adjustments according to both criteria. Furthermore, although ER and ERBW adjustments removed observed negative correlations of unadjusted analyte concentrations with UFR, they mostly resulted in positive correlations of an equal or greater magnitude. Excretion rate and ERBW adjustments thus performed no better than implementing no adjustment at all, with the sole exception of I, for which ERBW adjustment performed marginally better (*r*_p_ = 0.35 cf. -0.39).

**Fig. 4:**
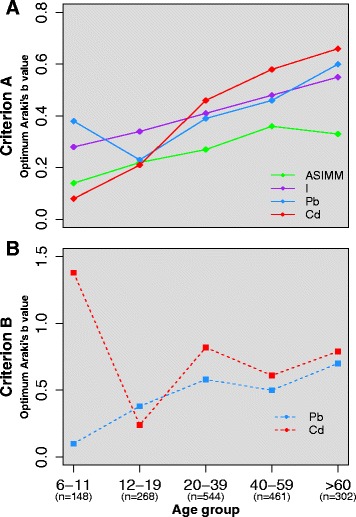
Optimum Criterion A (**a**) and Criterion B (**b**) Araki’s *b* values derived for different age groups for the range of analytes investigated

An exercise was undertaken to derive Araki’s *b* values for specific demographic groups of the data. The values derived for specific genders and ethnicities and specific age groups are presented in Table 7. Optimum Araki’s *b* values were plotted against age group for Criterion A (Fig. 4a) and Criterion B (Fig. 4b). Large differences in optimum *b* values were observed between different genders and ethnicities across the range of analytes but no obvious patterns were observed. For example, the optimum Araki’s *b* values for non-Hispanic white males were all lower than non-Hispanic white females for Criterion A but higher for Criterion B. For non-Hispanic black males, Araki’s *b* values were generally higher for both Criteria than for females. There was a general increase in optimum Araki’s *b* values with increasing age group across the range of analytes for both Criteria. In the case of Criterion A, for both gender/ethnicity and age groups, we attributed the difference in *b* values to the difference in group sizes. This is evident from the results presented in Table 7, where we also show the significance of the relationship between UFR and analyte concentrations. These slopes are synonymous with the optimum Criterion A values presented in Table 7 and, in smaller groups, some of the slopes are not significant. Nevertheless, Criterion B is independent of the relationship between UFR and analyte and, therefore, we chose to pursue the investigation of age-specific Araki’s *b* values for Criterion B. When UFRB adjustment was performed using *b* values specific to the volunteers’ age groups, no improvement in Criterion B correlations were observed relative to adjustment with a single group-wide *b* value (Pb: 0.73 versus 0.75; Cd: 0.60 versus 0.62).

**Table 7:** Araki’s *b* values derived for As^IMM^, I, Pb and Cd on specific demographic sub-groups of the present study group (training dataset)

Demographic group	*n*	Optimum Araki’s *b* value					
		Criterion A	Criterion A	Criterion A	Criterion A	Criterion B	Criterion B
		As^IMM^	I	Pb	Cd	Pb	Cd
Non-Hispanic white male	416	0.28***	0.50***	0.42***	0.31***	0.68	0.96
Non-Hispanic white female	425	0.33***	0.58***	0.50***	0.46***	0.54	0.63
Non-Hispanic black male	265	0.27***	0.51***	0.46***	0.29***	0.56	0.53
Non-Hispanic black female	224	0.24***	0.32***	0.24***	0.34***	0.44	0.53
Mexican American male	206	0.11*	0.30***	0.10	0.02	0.26	0.18
Mexican American female	187	0.34***	0.40***	0.46***	0.37***	0.58	0.56
6–11 years old	148	0.14*	0.28***	0.38***	0.08	0.10	1.38
12–19 years old	268	0.22***	0.34***	0.23***	0.21***	0.38	0.24
20–39 years old	544	0.27***	0.41***	0.39***	0.46***	0.58	0.82
40–59 years old	461	0.36***	0.48***	0.46***	0.58***	0.50	0.61
>60 years old	302	0.33***	0.55***	0.60***	0.66***	0.70	0.79

In the case of Criterion A, *** and * denote the significance of the relevant UFR-analyte regression slopes to *p* < 0.001 and 0.05, respectively

## Discussion

Osmolality and UFRA adjustment methods provided the best or near best performance of the adjustment methods tested using criterion A. Osmolality and creatinine adjusted concentrations yielded the strongest correlations using criterion B. In its nature, UFRA is tailored to optimise criterion A performance, however, osmolality and creatinine methods tended to perform better than Araki’s UFR-based adjustment methods with respect to criterion B. This may reflect greater uncertainties in volunteer reported times of initial voids than in uncertainties in objectively measured UFR surrogates such as osmolality or creatinine. The issue of reliance on the accuracy of volunteer reported void times when calculating UFR in NHANES has been raised previously [19]. We note this as a limitation of the present study, in that no efforts were made to refine UFR data or quantify their accuracy.

Alternatively, differences in Araki’s *b* values for given analytes, between individuals of different ages, genders and ethnicities may partly account for deficiencies in UFR based adjustments. Attempts were made to derive Araki’s *b* values on demographic subsets of the training dataset. Differences in optimum values were observed between groups but this was possibly an artefact of different group sizes. When age-specific *b* values were implemented to the adjustment of urinary Pb and Cd, no significant improvements were observed in Criterion B correlations. Future efforts should be made to derive Araki’s *b* values for multiple individuals, collecting multiple voids at various states of hydration, to more closely represent the relationship between analyte concentrations and UFR, as illustrated in Fig. 1f. As proposed previously [23], these derivations should be made on specific demographic groups to investigate whether the relationship between analyte concentrations and UFR vary in a characteristic manner, something which was not achievable given the constraints of the data utilised in the present study.

The finding that Araki’s *b* values calculated here by the UFRA method were generally lower than those calculated previously [20, 21], might give rise to questioning of the validity of the UFRA based values. We therefore identify a potential flaw in the validity of criterion A when using the present dataset. As discussed, it was not possible to derive Araki’s *b* values in the conventional manner using the NHANES dataset. This requires multiple voids from single volunteers at different hydration states, circumstances under which the ‘true’ relationship between UFR and analyte concentration is observed due to the relatively constant internal dose of a given analyte, for a single individual, over the timescale investigated. NHANES data consist of single voids from multiple individuals with greater inter-individual ranges of internal doses which have the potential to alter the observed slope (*b* value). Under circumstances where the standard deviation of the distribution of internal dose is considerably bigger than that of the distribution of UFR for the studied population, the calculated Araki’s *b* value may be positively biased. This in-turn determines the optimum value for criterion A - the *b* value that describes the slope between UFR and analyte – evident in the agreement between the *b* values that describe the slopes presented in Fig. 1 and those derived using the numeric method. This is further illustrated by the difference between criteria A and B optimum values for Pb and Cd (Fig. 2a and b), the criterion B optimums could be considered more robust for the NHANES dataset used here as they are independent of UFR. We note, however, that this paper does not purport to suggest specific Araki’s *b* values for application elsewhere, but is successful in reiterating proof of concept of their necessary implementation to remove hydration variation from spot analyte concentrations and, in doing so (Fig. 2a and b), better reflect internal dose.

Our findings reiterate the analyte-specific nature of hydration adjustment, exemplified by the difference in criteria A correlations between creatinine adjusted As^IMM^ and I. Creatinine adjusted I concentrations yielded no significant correlation with UFR, whereas creatinine adjusted As^IMM^ concentrations were more strongly correlated with UFR than I, Pb and Cd. Inspection of Fig. 1c indicates differing relationships with UFR of urinary As^IMM^ for high-DMA and low-DMA samples – this suggests that the biochemistry of different species of the same element (and different elements) influence Araki’s *b* values.

As noted previously [19], for studies outside the NHANES framework or similar population-scale biomonitoring designs, the collection of UFRs may provide additional value providing that accurate recordings of time and volume are obtained. It is recognised that this may not be logistically feasible for all studies and surrogates such as creatinine and osmolality are attractive alternatives. Modifications of these surrogate based adjustments have also been explored [22, 23, 42] using methodologies based on the work of Araki et al. (1986). These approaches, such as modified SG [23] or modified creatinine [25] adjustment, were not addressed in the present study and further exploration of such alternatives may prove valuable for studies that are restricted in their ability to directly measure UFR, particularly in low-budget circumstances or developing countries. Similarly, measurements of additional urinary constituents that are indicative of medical conditions, such as glucose, protein, ketones and bilirubin, could have provided additional data exclusion criteria had they been available to us. A comparison of the performance of different adjustment methods between volunteers with and without the presence of such analytes, and the medical conditions that were available as exclusion criteria (e.g. CKD and diabetes) will make for an important matter of further research.

The implications of the findings made in this investigation, and the questions that remain unanswered, have implications for environmental and epidemiological studies using urinary biomonitoring to assess human exposures and investigate the dose-response relationships between environmental chemicals and health end-points. The differences in biomarker levels yielded by different adjustment methods is evident (Table 4). This impacts the interpretation of results when making comparisons to existing guidance or reference values. Furthermore, it impacts the derivation of reference values themselves. For example, a large and much needed body of work has been undertaken to derive biomonitoring equivalents to be used in comparison with various urinary biomarkers [43]. Some derivations have utilised creatinine adjustment, which may have limited their applicability to studies using alternative adjustment methods, or other studies using creatinine adjustment with different demographic structures. This problem also extends to studies that explore relationships between urinary analyte concentrations and health outcomes- a widely used application of NHANES data [44–46]. A robust, standardised framework of urinary hydration adjustment is warranted to ensure the validity and sensitivity of such analyses.

## Conclusions

We have demonstrated for the urinary analytes studied (Pb; Cd; As^IMM^ and I), and the adjustment methods considered (osmolality; creatinine; excretion rate (ER); body weight adjusted excretion rate (ERBW); urinary flow rate adjustment with Araki’s *b* optimised to minimise correlation with urinary flow rate (UFRA) and urinary flow rate adjustment with Araki’s *b* optimised to maximise correlation with blood analyte concentrations (UFRB):

(i) Osmolality consistently performs as the best or near best adjustment method against two performance criteria: minimum correlation of adjusted urinary analyte concentration with UFR; maximum correlation of adjusted urinary analyte concentration with blood analyte concentration.
(ii) The method of Araki et al. (1986, 1990) for objectively determining Araki’s *b* values to adjust urinary analyte concentrations also performs well, but is limited by the requirement for accurately determined UFR data and age/gender/ethnicity specific *b* values.
(iii) Creatinine adjustment methods may be suitable for some analytes (e.g. I) that have similar *b* values to creatinine, but can otherwise result in significant biases.
(iv) ER and ERBW based adjustment methods are shown here to overcompensate for UFR and invariable performed worse than osmolality, creatinine, UFRA, UFRB and often worse than unadjusted concentrations.

Thus, we demonstrate that conventional application of UFR is limiting the full potential of this metric. The under-performance of both ER and ERBW in relation to two independent performance criteria support previous findings [20, 21] that using UFR to calculate excretion rates in the conventional manner propagates inaccurate results. The inclusion of Araki’s *b* values into adjustment calculations was demonstrated to significantly improve the performance of UFR adjustment for both criteria relative to ER and ERBW adjustments.

The derivation of specific Araki’s *b* values requires substantial further work by collecting multiple voids from the same individuals. By compiling a range of Araki’s *b* values for a range of analytes and demographic characteristics, their determining factors can be assessed, enabling the development of more sophisticated framework for urinary biomarker adjustment. Finally, additional constraints on the interpretation of urinary biomarker concentrations need addressing, such as time of sampling relative to exposure [1], which hydration adjustment cannot overcome.

## Abbreviations

As^III^, arsenous acid; As^IMM^, Inorganic arsenic and Methylated Metabolites; As^V^, arsenic acid; BW, bodyweight; CI, confidence interval; CKD, chronic kidney disease; DMA, dimethylarsonic acid; eGFR, estimated glomerular filtration rate; ER, excretion rate; ERBW, excretion rate adjusted for body weight; FMV, first morning void; GM, geometric mean; HPLC, high performance liquid chromatography; ICP-DRC-MS, inductively coupled plasma dynamic reaction cell mass spectrometry; MEC, Mobile Examination Center; MMA, monomethylarsonic acid; NHANES, National Health and Nutrition Examination Survey; r_p_, Pearson’s correlation coefficient; r_s_, Spearman’s correlation coefficient; SG, Specific Gravity; UFR, urinary flow rate; UFRA, urinary flow rate adjustment optimised to criterion A; UFRB, urinary flow rate adjustment optimised to criterion B

## Additional file

Additional file 1: Supporting R programming script for the numerical derivation of opimum Araki's b values and adjustment of urinary analyte concentrations using the methods described in this paper. (R 8 kb)

## References

[1] Aylward LL, Hays SM, Smolders R, Koch HM, Cocker J, Jones K (2014). Sources of variability in biomarker concentrations. J Toxicol Environ Health B Crit Rev.

[2] Middleton DRS, Watts MJ, Hamilton EM, Ander EL, Close RM, Exley KS, Crabbe H, Leonardi GS, Fletcher T and Polya D.A. 2016. Urinary arsenic profiles reveal substantial exposures to inorganic arsenic from private drinking water supplies in Cornwall, UK, Sci Rep, DOI: SREP25656. 10.1038/srep25656 PMC4860641 27156998

[3] Watts M, Joy E, Young S, Broadley M, Chilimba A, Gibson R (2015). Iodine source apportionment in the Malawian diet. Sci Rep.

[4] Cone EJ, Caplan YH, Moser F, Robert T, Shelby MK, Black DL (2009). Normalization of urinary drug concentrations with specific gravity and creatinine. J Anal Toxicol.

[5] Moeller KE, Lee KC, Kissack JC. Urine drug screening: Practical guide for clinicians. In: Proceedings of the Mayo Clinic Proceedings. Amsterdam, Netherlands: 2008;83: 66–76. 10.4065/83.1.66 18174009

[6] Sobus JR, DeWoskin RS, Tan Y-M, Pleil JD, Phillips MB, George BJ (2015). Uses of NHANES biomarker data for chemical risk assessment: Trends, challenges, and opportunities. Environ Health Perspect.

[7] Barrett JR (2015). Urinary biomarkers as exposure surrogates: Controlling for possible bias. Environ Health Perspect.

[8] Yeh HC, Lin YS, Kuo CC, Weidemann D, Weaver V, Fadrowski J (2015). Urine osmolality in the US population: Implications for environmental biomonitoring. Environ Res.

[9] Rivera-Núñez Z, Meliker JR, Linder AM, Nriagu JO (2010). Reliability of spot urine samples in assessing arsenic exposure. Int J Hyg Environ Health.

[10] Barr DB, Wilder LC, Caudill SP, Gonzalez AJ, Needham LL, Pirkle JL (2005). Urinary creatinine concentrations in the US population: Implications for urinary biologic monitoring measurements. Environ Health Perspect.

[11] Mayersohn M, Conrad KA, Achari R (1983). The influence of a cooked meat meal on creatinine plasma concentration and creatinine clearance. Br J Clin Pharmacol.

[12] Baxmann AC, Ahmed MS, Marques NC, Menon VB, Pereira AB, Kirsztajn GM (2008). Influence of muscle mass and physical activity on serum and urinary creatinine and serum cystatin C. Clin J Am Soc Nephrol.

[13] Nermell B, Lindberg AL, Rahman M, Berglund M, Åke Persson L, El Arifeen S (2008). Urinary arsenic concentration adjustment factors and malnutrition. Environ Res.

[14] Barber T, Wallis G (1986). Correction of urinary mercury concentration by specific gravity, osmolality, and creatinine. J Occup Environ Med.

[15] Leech S, Penney M (1987). Correlation of specific gravity and osmolality of urine in neonates and adults. Arch Dis Child.

[16] Dossin O, Germain C, Braun JP (2003). Comparison of the techniques of evaluation of urine dilution/concentration in the dog. J Vet Med A Physiol Pathol Clin Med.

[17] Imran S, Eva G, Christopher S, Flynn E, Henner D (2010). Is specific gravity a good estimate of urine osmolality?. J Clin Lab Anal.

[18] Sauvé JF, Lévesque M, Huard M, Drolet D, Lavoué J, Tardif R (2015). Creatinine and specific gravity normalization in biological monitoring of occupational exposures. J Occup Environ Hyg.

[19] Hays SM, Aylward LL, Blount BC (2015). Variation in urinary flow rates according to demographic characteristics and body mass index in NHANES: potential confounding of associations between health outcomes and urinary biomarker concentrations. Environ Health Perspect.

[20] Araki S, Murata K, Aono H, Yanagihara S, Niinuma Y, Yamamoto R (1986). Comparison of the effects of urinary flow on adjusted and non‐adjusted excretion of heavy metals and organic substances in ‘healthy’ men. J Appl Toxicol.

[21] Araki S, Sata F, Murata K (1990). Adjustment for urinary flow rate: an improved approach to biological monitoring. Int Arch Occup Environ Health.

[22] Sorahan T, Pang D, Esmen N, Sadhra S (2008). Urinary concentrations of toxic substances: an assessment of alternative approaches to adjusting for specific gravity. J Occup Environ Hyg.

[23] Vij HS, Howell S (1998). Improving the specific gravity adjustment method for assessing urinary concentrations of toxic substances. Am Ind Hyg Assoc J.

[24] CDC (Centers for Disease Control and Prevention). 2013. National Biomonitoring Program Lead Biomonitoring Summary Webpage. Available: http://www.cdc.gov/biomonitoring/Lead_BiomonitoringSummary.html [Accessed 05 Sep 2015].

[25] Sata F, Araki S, Yokoyama K, Murata K. Adjustment of creatinine-adjusted values in urine to urinary flow rate: a study of eleven heavy metals and organic substances. Int Arch Occup Environ Health. 1995;68:64-68. 10.1007/BF01831635 8847115

[26] CDC (Centers for Disease Control and Prevention). 2013. National Biomonitoring Program Cadmium Biomonitoring Summary Webpage. Available: http://www.cdc.gov/biomonitoring/Cadmium_BiomonitoringSummary.html [Accessed 05 Sep 2015].

[27] Akerstrom M, Barregard L, Lundh T, Sallsten G (2013). The relationship between cadmium in kidney and cadmium in urine and blood in an environmentally exposed population. Toxicol Appl Pharmacol.

[28] Middleton D, Watts M, Hamilton E, Fletcher T, Leonardi G, Close R (2016). Prolonged exposure to arsenic in UK private water supplies: Toenail, hair and drinking water concentrations. Env Sci Process Impact.

[29] CDC (Centers for Disease Control and Prevention). 2015. National Health and Nutrition Examination Survey Homepage. Available: http://www.cdc.gov/nchs/nhanes.htm [Accessed 01 Oct 2015].

[30] Warnes GR. 2014. Sasxport: Read and write sas xport files. https://cran.r-project.org/web/packages/SASxport/index.html.

[31] Dragulescu AA. 2014. Xlsx: Read, write, format excel 2007 and excel 97/2000/xp/2003 files. https://cran.r-project.org/web/packages/xlsx/index.html.

[32] Levey AS, Stevens LA, Schmid CH, Zhang YL, Castro AF, Feldman HI (2009). A new equation to estimate glomerular filtration rate. Ann Intern Med.

[33] Schwartz GJ, Muñoz A, Schneider MF, Mak RH, Kaskel F, Warady BA (2009). New equations to estimate GFR in children with CKD. J Am Soc Nephrol.

[34] Meharg A, Williams P, Deacon C, Norton G, Hossain M, Louhing D (2014). Urinary excretion of arsenic following rice consumption. Environ Pollut.

[35] R Core Team. 2013. R: A language and environment for statistical computing, r foundation for statistical computing, Vienna, Austria, www.R-project.Org.

[36] Kuhn M. 2015. Caret: Classification and regression training. https://cran.r-project.org/web/packages/caret/index.html.

[37] Dobbin KK, Simon RM (2011). Optimally splitting cases for training and testing high dimensional classifiers. BMC Med Genomics.

[38] Levine L, Fahy JP (1945). Evaluation of urinary lead concentrations. 1. The significance of the specific gravity. J Ind Hyg Toxicol.

[39] Wilcox RR, Tian T (2008). Comparing dependent correlations. J Gen Psychol.

[40] Revelle W. 2014. Psych: Procedures for psychological, psychometric, and personality research, Northwestern University, Evanston, Illinois, https://cran.r-project.org/web/packages/psych/index.html.

[41] Venables WNR, Ripley BD (2002). Modern applied statistics with S.

[42] Sata F, Araki S (1996). Adjustment of creatinine-adjusted value to urine flow rate in lead workers.

[43] Hays S, Becker R, Leung H, Aylward L, Pyatt D (2007). Biomonitoring equivalents: a screening approach for interpreting biomonitoring results from a public health risk perspective. Regul Toxicol Pharmacol.

[44] Navas-Acien A, Silbergeld EK, Pastor-Barriuso R, Guallar E (2008). Arsenic exposure and prevalence of type 2 diabetes in US adults. JAMA.

[45] Tellez-Plaza M, Navas-Acien A, Crainiceanu CM, Guallar E. 2008. Cadmium exposure and hypertension in the 1999-2004 national health and nutrition examination survey (NHANES). Environ Health Perspect: 116:51-56. 10.1289/ehp.10764 PMC2199293 18197299

[46] Haddow JE, McClain MR, Palomaki GE, Hollowell JG (2007). Urine iodine measurements, creatinine adjustment, and thyroid deficiency in an adult United States population. J Clin Endocrinol Metab.

